# Comparative effects of dietary pomegranate peel and *Aloe vera* gel on growth, metabolic pathways, antioxidant status, molecular docking, and intestinal integrity in growing rabbits

**DOI:** 10.3389/fnut.2026.1750178

**Published:** 2026-02-19

**Authors:** Mohsen A. Khormi, Seham Samir Soliman, Sameh A. Abdelnour, Manal R. Bakeer

**Affiliations:** 1 Department of Biology, College of Science, Jazan University, Jazan, Saudi Arabia; 2 Department of Animal Reproduction and Artificial Insemination, Veterinary Research Institute, National Research Centre (NRC), Dokki, Cairo, Egypt; 3 Department of Animal Production, Faculty of Agriculture, Zagazig University, Zagazig, Egypt; 4 Department of Physiology, Faculty of Veterinary Medicine, Cairo University, Giza, Egypt

**Keywords:** Acemannan, *Aloe vera*, antioxidant activity, growth performance, molecular docking, *Oryctolagus cuniculus*, *Punica granatum*, punicalagin

## Abstract

**Background:**

*Aloe vera* gel is rich in polysaccharides (acemannan), phenolic compounds, vitamins, and minerals, while Pomegranate peel (*Punica granatum* L.) is a valuable source of punicalagin, polyphenols, tannins, flavonoids, dietary fiber, vitamins, and minerals. This study examined the impact of these bioactive-rich supplements on growth performance, metabolic activity, digestive enzyme function, antioxidant status, levels of nucleic acids and proteins, as well as gastrointestinal histomorphometry in growing rabbits.

**Materials and methods:**

Thirty male New Zealand White rabbits (56 ± 3 days) at the start of the trial, corresponding to the early post-weaning/growing phase in New Zealand White rabbits. Rabbits were randomly divided into three groups (*n* = 10) and treated for 14 weeks. The control group (C) received a basal diet; the pomegranate group (P) received the basal diet supplemented with 4.5% pomegranate peel; and the *Aloe vera* group (A) received the basal diet with drinking water containing *Aloe vera* gel (500 mg/L).

**Results:**

Both supplemented groups exhibited significantly higher body weight and improved metabolic indices, including elevated blood glucose, total protein and lipid profile, compared with the control group (*p* < 0.05). Activities of amylase, lipase, and protease increased significantly, with stronger stimulation observed in the pomegranate group. Antioxidant assays revealed higher total antioxidant capacity (TAC) and catalase (CAT) activity, accompanied by reduced malondialdehyde (MDA) levels in both supplemented groups (*p* < 0.05). DNA and total protein concentrations were also elevated, particularly in the pomegranate group. Histomorphometric analysis of the duodenum showed significant improvements in villus height, crypt depth, and glandular area (*p* < 0.05). *Aloe vera* supplementation exerted greater effects on villus and crypt architecture, whereas pomegranate peel predominantly enhanced glandular development. Molecular docking simulations revealed that acemannan and punicalagin possess high binding affinities for pro-apoptotic and antioxidant targets. Specifically, acemannan exhibited markedly lower binding energies than punicalagin for both BAX (−10.627 vs. –7.540 kcal/mol) and SOD (−10.544 vs. –7.663 kcal/mol). These results suggest that acemannan may exert superior bioactivity by effectively modulating BAX-mediated apoptosis and augmenting SOD-driven antioxidant defense through stable protein-ligand complexation.

**Discussion:**

In conclusion, dietary supplementation with pomegranate peel or *Aloe vera* significantly improved growth performance, optimized metabolic activity, and enhanced intestinal morphology in growing rabbits. Each supplement provided unique physiological benefits, supported by molecular docking evidence linking their bioactive compounds to antioxidant and cytoprotective mechanisms.

## 1 Introduction

Global food production faces considerable challenges due to losses and waste, estimated at approximately 1.3 billion tons per year, with an associated economic burden of nearly USD 990 billion. The fruit and vegetable processing sectors are a major contributor to this issue, generating up to 45% of total by-products. When mismanaged, these materials represent both a loss of valuable nutritional resources and a significant environmental threat (1).

Rabbits are increasingly vital to sustainable livestock systems, valued for their rapid reproductive cycles, superior feed conversion efficiency, and cost-effectiveness (2). Beyond production advantages, rabbit meat serves as a functional food, offering high protein levels and a lean lipid profile enriched with essential amino acids, minerals, and vitamins (3). However, the weaning period remains a pivotal challenge; during this stage, kits are highly susceptible to environmental, social, and nutritional stressors (4). Consequently, precision nutrition is vital to sustain growth trajectories and safeguard intestinal integrity (3).

Agro-industrial by-products, often disposed of improperly, are rich in bioactive compounds that can mitigate oxidative stress and promote health (5, 6). Utilizing these by-products as feed ingredients offers economic and environmental benefits and is an increasingly important approach in sustainable livestock production (7).

Pomegranate (*Punica granatum* L.), cultivated since around 3,000 B.C. in regions such as Iran, India, China, and the Mediterranean, is now grown globally (8). The fruit comprises peel, juice, and seeds. Juice production generates substantial peel waste, representing 26%–30% of the fruit’s weight. Pomegranate peel is rich in polyphenols, fiber, vitamins, and minerals (9), with documented antioxidant, anti-inflammatory, and anticancer properties (10). Extracts have shown potential in preventing or alleviating metabolic disorders such as diabetes, obesity, and cardiovascular disease (11). Although pomegranate extracts have been explored in rabbit production to improve growth and nutrient digestibility (12), the direct use of raw pomegranate peels in rabbit diets during the weaning stage remains underexplored.

*Aloe vera* (L.) Webb, also known as *Aloe barbadensis* Mill., is a drought-tolerant succulent native to Eastern Africa, India, China, and the Mediterranean. It is now cultivated in many regions including Malta, India, and the USA. The thick leaves of *Aloe vera* contain a mucilaginous gel rich in anthraquinones, lectins, glucomannans, fatty acids, sterols, tannins, enzymes, and minerals (13). Traditionally, *Aloe vera* is considered a natural phytogenic supplement with therapeutic value. It has been used for burns, wounds, gastrointestinal disorders, and skin diseases due to its anti-inflammatory, antibacterial, and wound-healing properties (14).

Given the rising interest in sustainable feed additives derived from agro-industrial by-products (15, 16), this study aimed to evaluate the effects of dietary supplementation with pomegranate peel and *Aloe vera* on gastrointestinal enzyme activity, antioxidant and biochemical parameters, molecular docking analysis, DNA and protein concentrations, and histomorphometric characteristics in the gastrointestinal tract of growing rabbits.

## 2 Materials and methods

The study was conducted in the experimental pen of the Faculty of Veterinary Medicine, Cairo University. The protocol for the experiment was approved by the Institutional Animal Care and Use Committee (IACUC) of the Faculty of Veterinary Medicine, Cairo University, Giza, Egypt, under reference number Vet CU 09092023781.

### 2.1 Experimental design

A total of 30 clinically healthy male New Zealand White rabbits (*Oryctolagus cuniculus*), aged 56 ± 3 days at the start of the trial and with an average initial body weight of approximately 1,000 g ± 22 g, were randomly divided into three experimental groups, with 10 rabbits in each group. Before the feeding trial, all rabbits underwent a 7-day adaptation period to adjust to their diets and housing conditions.

The experimental groups were as follows:

- Control group (C): Rabbits received a standard basal diet without any supplementation.
- Pomegranate group (P): Rabbits received the basal diet supplemented with 4.5% pomegranate peel powder, as per the method described by Zeweil and Elgindy (17).
- *Aloe vera* group (A): Rabbits were provided with *Aloe vera* gel in their drinking water at a concentration of 500 mg/L, following the protocol outlined by Channa et al. (18).

All animals were housed under standard environmental conditions and had *ad libitum* access to feed and water throughout the experimental period. A dose of 4.5% pomegranate peel was chosen based on previous rabbit studies where this inclusion level improved performance and antioxidant status without causing adverse effects on feed intake or digestibility (17). The 500 mg/L *Aloe vera* gel concentration was selected according to Channa et al. (18) and subsequent reports indicating its efficacy in enhancing growth and immune responses in rabbits.

### 2.2 *Aloe vera* gel preparation

Mature, healthy *Aloe vera* (*Aloe barbadensis* Miller) leaves, measuring 25–50 cm in length, were collected fresh from El-Hossary Garden plantations and taxonomically identified by a botanist. This species belongs to the Liliaceae family, and is widely recognized as the most bioactive Aloe species. The harvested leaves were thoroughly washed with clean water and dried using a sterile cloth. The basal 25 mm white portion attached to the rosette stem, the apical 50–100 mm tapering end, and the marginal spines were removed using a sharp, sterile knife. The cleaned leaves were then longitudinally split, and the inner white pulp was scraped and weighed. The central gel was carefully extracted and homogenized using a blender. Fresh gel was prepared before each administration and administered with drinking water, following the protocol described by Oyewopo et al. (19).

### 2.3 Animal housing and rearing

The rabbits were housed individually in wire cages equipped with J-feeders and automatic nipple drinking systems to ensure they had ad libitum access to feed and water. Each cage measured 80 × 60 × 45 cm (4,800 cm^2^ floor area), which meets the recommended minimum space requirements for laboratory rabbits according to the NRC (8th Edition) and the Guide for the Care and Use of Laboratory Animals (8th Edition, National Academies Press). The experimental facility was well-ventilated, with electric fans and operable windows, and was illuminated using a combination of natural and fluorescent lighting on a 14:10 h light/dark cycle. Environmental conditions were maintained at approximately 75% relative humidity and an ambient temperature of around 25 °C. All rabbits underwent clinical health screening by a licensed veterinarian before the experiment. The animals were confirmed to be in good health based on physical examination, normal behavior, and the absence of external or internal parasitic signs. Two weeks before the experiment, the rabbits were dewormed with ivermectin (0.2 mg/kg, subcutaneously). No vaccinations were given during the experimental period, as the rabbits were kept in controlled institutional conditions with strict biosecurity measures.

Diets for both the control and experimental groups were formulated based on the nutritional requirements of rabbits, as recommended by the National Research Council (20). All animals had *ad libitum* access to their respective diets. The feed underwent proximate chemical analysis according to the standard procedures of the Association of Official Analytical Chemists (21). The ingredient composition and proximate chemical analysis of the formulated diets used in this study are presented in Table 1.

**Table 1:** Composition percentage and nutrient profile of the basal and experimental diets.

Ingredients %	Control	Pomegranate peel (P)	*Aloe vera* (A)
Berseem hay	30.00	29.10	30.00
Pomegranate peel	–	4.50	–
*Aloe vera*	–	–	500 mg*
Barley grain	21.00	21.10	21.00
Yellow corn	5.00	3.00	5.00
Wheat bran	21.10	24.40	21.10
Soybean meal	17.50	12.50	17.50
Molasses	3.00	3.00	3.00
CaCl₂	1.50	1.50	1.50
NaCl	0.40	0.40	0.40
Vitamin & mineral premix	0.30	0.30	0.30
DL-methionine	0.20	0.20	0.20
Chemical compositions%			
Moisture	9.40	9.50	9.40
Dry matter	90.60	90.50	90.60
Crude protein	17.50	17.46	17.50
Crude fiber	16.00	16.41	16.00
Ether extract	2.53	2.39	2.53
Total ash	7.10	7.18	7.10
Nitrogen-free extract (NFE)	47.47	47.06	47.47
Digestible energy (kcal/kg)	2698.89	2698.41	2698.89

Vitamin and mineral premix (per kg diet): Vitamin A, 4,000,000 IU; Vitamin D₃, 500,000 IU; Vitamin E, 16.7 g; Vitamin K, 0.67 g; Vitamin B₁, 0.67 g; Vitamin B₂, 2 g; Vitamin B₆, 0.67 g; Vitamin B₁₂, 0.004 g; Vitamin B₅, 16.7 g; Pantothenic acid, 6.67 g; Biotin, 0.07 g; Folic acid, 1.67 g; Choline chloride, 400 g; Zn, 23.3 g; Mn, 10 g; Fe, 25 g; Cu, 1.67 g; I, 0.25 g; Se, 0.033 g; Mg, 133.4 g. *The *Aloe vera* (A) group received the same basal diet as the control group; *Aloe vera* gel was administered via drinking water at a concentration of 500 mg/L.

### 2.4 Sampling and measured parameters

#### 2.4.1 Blood samples

At the end of the experiment, blood samples were collected from the marginal ear vein in the fasted state after 12 h without feed, immediately prior to morning feeding/supplementation, to ensure consistent metabolic baseline conditions across all groups. The blood was allowed to coagulate for 2 hours at room temperature before being centrifuged at 860 × *g* for 20 min to separate the serum. Serum glucose levels were measured immediately after collection. The remaining serum was then aliquoted and stored at −20 °C for later analysis of metabolic parameters, digestive enzymes, and antioxidant markers. All biochemical parameters were estimated using commercially available kits from Spinreact, S.A.U., Girona, Spain. Furthermore, the final body weights of all rabbits in each group were recorded using an electronic digital balance.

#### 2.4.2 Metabolic parameters

Glucose concentration was determined using the enzymatic oxidase–peroxidase method (22). Total protein content was quantified by the dye-binding method (23), and total lipid concentration was determined using the enzymatic colorimetric technique (24).

#### 2.4.3 Digestive enzyme activities

Amylase activity in serum was estimated using the method described by Ibrahim (25). Lipase activity was measured according to the technique of Wang (26), while protease activity was determined following the protocol established by Guo (27).

#### 2.4.4 Antioxidant parameters

Total antioxidant capacity (TAC), catalase (CAT) activity, and lipid peroxidation, expressed as malondialdehyde (MDA), were determined using commercially available kits from Bio diagnostic, Giza, Egypt (28).

#### 2.4.5 DNA and protein quantification in duodenum

The experimental period lasted 14 weeks, during which all rabbits were slaughtered and eviscerated. Tissue samples were collected from the duodenum, rapidly frozen in liquid nitrogen (−196 °C), and stored for subsequent analyses, including DNA and protein quantification, as well as histological examination. Protein extraction was performed from homogenized duodenal tissue using phosphate-buffered saline, followed by centrifugation, and the total protein concentration was determined using the Bradford colorimetric method (23). DNA extraction was conducted using the QIAamp Mini DNA Purification Kit (Qiagen, Germany), and DNA concentration and purity were quantified spectrophotometrically at absorbance of 260/280 nm using a NanoDrop 2000 (Thermo Fisher Scientific, USA).

#### 2.4.6 Histomorphometry examination

Tissue specimens were processed for paraffin embedding to facilitate micromorphological examination. Standard histological procedures were followed, including rinsing in tap water, fixation in 10% neutral buffered formalin (NBF), dehydration through a graded alcohol series, clearing in xylene, and embedding in paraffin wax. Sections were cut at a thickness of 3–5 μm using a microtome, followed by deparaffinization and staining. Hematoxylin and eosin (H&E) staining was used to examine general tissue architecture, while Alcian blue staining was applied to visualize mucous secretions in the submucosal glands of the duodenum (29). The stained sections were examined under a light microscope (LEICA DM500, Leica Microsystems, Wetzlar, Germany) Digital images were captured with a camera (LEICA ICC50 HD, Leica Microsystems, Wetzlar, Germany) attached to the microscope and analyzed with image analysis software (Leica Microsystems, LAS version 3.8.0 [build: 878], Leica Ltd., Wetzlar, Germany) (30). Number of animals analyzed per group (*n* = 4). Number of sections analyzed per animal (3 non-adjacent sections). Number of villi and crypts measured per section (at least 10). Section spacing and standardized field selection criteria. Measurements were performed by an investigator blinded to treatment groups.

#### 2.4.7 *In silico* analysis

Both *Aloe vera* (Acemannan) and pomegranate peel (punicalagin) were subjected to an active site molecular docking simulation to investigate their biological activities. These processes were executed using the Molecular Operating Environment (MOE 2014.10, Chemical Computing Group, Montreal, QC, Canada) software. The selected proteins were apoptosis regulator BAX, coded G1SQZ2, and extracellular superoxide dismutase, SOD, coded P41975, which were downloaded from the UniProt database.^1^ The 2D structures of the ligands were obtained from the PubChem database^2^ in SDF file formats. The protein pretreatments involved the elimination of all associated ligands while retaining the cofactors to replicate their biological functions within the body using Biovia Discovery studio software. AutoDock 4.2 software was managed the molecular docking processes during this investigation. Polar hydrogens were incorporated into the proteins, followed by the integration of Kollman charges and the assignment of Gasteiger. The grid specifications were established at 126 × 126 × 126 points with a spacing of 1.000 Å to cover the whole body of the protein in order to proceed a blind molecular docking simulation. The OpenBabel program was used for file format conversion into MOL extensions. The ligands were cleaned and their energies minimized before entering the docking procedures. Both ligands were saved in one established database to undergo the same process of molecular docking with the same active site (dummy atoms). The protein preparation stage on the MOE software began by correcting the structure and 3D protonation, as well as minimizing the total energy. The site finder was used to determine the active site as dummy atoms. The docking stage proceeded with 100 retries to ensure accurate results of the binding affinities/scores and incoming non-covalent interactions of the ligand-protein complexations.

### 2.5 Statistical analysis

Data were analyzed using one-way analysis of variance (ANOVA) in SAS software. (Version 9.4, SAS Institute Inc., Cary, NC, USA). Differences between group means were assessed using Tukey’s *post hoc* test. The significance level of *p* < 0.05 was considered statistically significant. Results are presented as mean ± standard error of the mean (SEM). The dataset was tested for normal distribution using the Shapiro–Wilk test and for homogeneity of variances using Levene’s test. The results confirmed that the data met the assumptions of parametric analysis.

## 3 Results

### 3.1 Body weight and metabolic analysis

**Figure 1:**
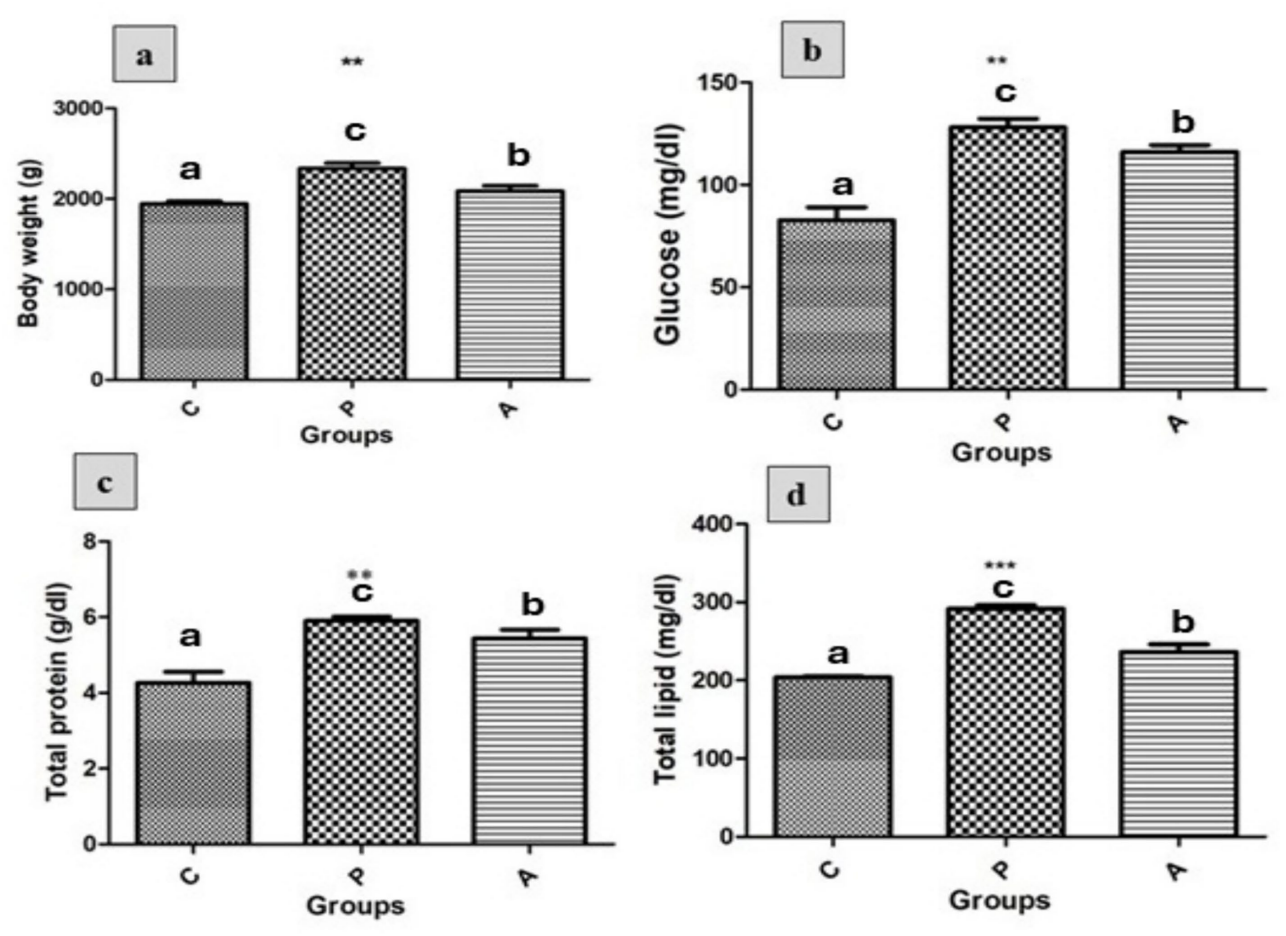
The effects of different treatments on **(a)** body weight (g), **(b)** plasma glucose concentration (mg/dL), **(c)** total protein concentration (g/dL), and **(d)** total lipid concentration (mg/dL) in growing rabbits across experimental groups. Group C = Control; Group P = Pomegranate; Group A = *Aloe vera*, *n* = (10). Data are presented as mean ± standard error (SEM). Asterisks denote significant differences compared to the control group (*p < 0.05, **p < 0.01, ***p < 0.001). Different letters indicate significant differences between experimental groups (*p* < 0.05).

Body weight and metabolic parameters were assessed in all treatment groups (Figure 1). A significant increase in body weight (Figure 1a, *p* < 0.01), as well as in metabolic indicators including blood glucose (Figure 1b, *p* < 0.01), total protein (Figure 1c, *p* < 0.01), and total lipid concentrations (Figure 1d, *p* < 0.001) was observed in both the pomegranate peel supplemented group (P) and the *aloe vera*-treated group (A) compared with the control group (C) (*p* < 0.05). Notably, the P group exhibited a slightly greater increase across all four parameters than the A group (*p* < 0.05).

### 3.2 The activity of digestive enzymes

**Figure 2:**
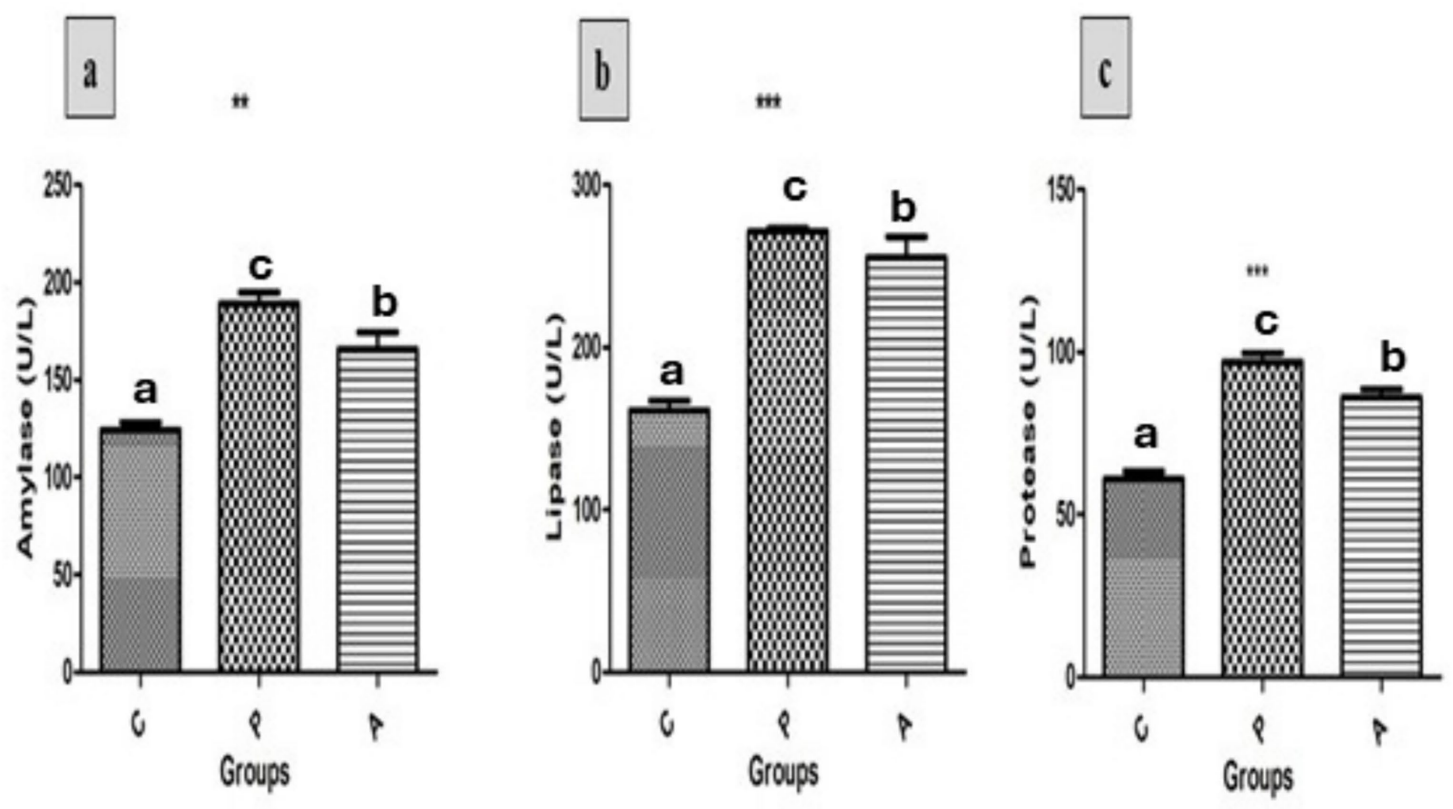
Effect of treatment on the activity of key digestive enzymes: **(a)** amylase, **(b)** lipase, and **(c)** protease in growing rabbits across experimental groups. Group C = control; Group P = pomegranate; Group A = *Aloe vera, n* = (10). Data are presented as mean ± standard error (SEM). Asterisks denote significant differences compared to the control group (*p < 0.05, **p < 0.01, ***p < 0.001). Different letters indicate significant differences between experimental groups (*p* < 0.05).

The present study assessed the activity of various digestive enzymes across all treatment groups (Figure 2). Significant increases in amylase (Figure 2a), lipase (Figure 2b), and protease (Figure 2c) activities were observed in both the pomegranate peel-supplemented group (P) and the *aloe vera*-supplemented group (A), compared to the control group (C) (*p* < 0.05). Notably, the P group exhibited significantly higher enzyme activity levels than both the A and C groups.

### 3.3 Antioxidants analysis

**Figure 3:**
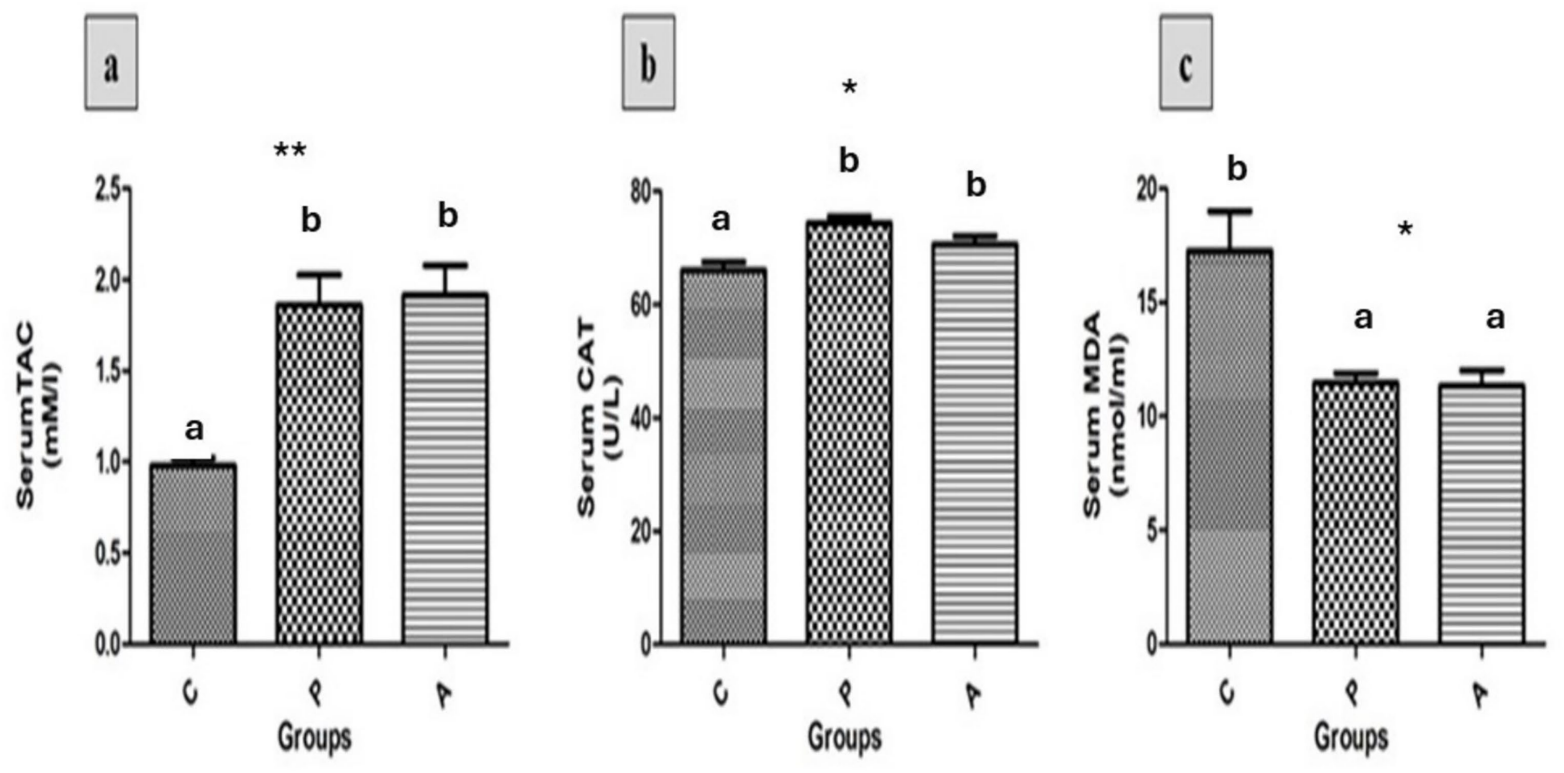
The effects of different treatments on serum total antioxidant capacity (TAC, **a**), catalase activity (CAT, **b**), and malondialdehyde (MDA, **c**) levels in growing rabbits across experimental groups. Group C = control; Group P = pomegranate; Group A = *Aloe vera*, *n* = (10). Asterisks denote significant differences compared to the control group (*p < 0.05, **p < 0.01, ***p < 0.001). Different letters indicate significant differences between experimental groups (*p* < 0.05).

Serum total antioxidant capacity (TAC), catalase (CAT), and malondialdehyde (MDA) levels were assessed across all experimental groups (Figure 3). The results demonstrated a significant increase in serum TAC (Figure 3a, *p* <0.01) and CAT activity (Figure 3b, *p* < 0.05) in both the pomegranate peel-supplemented group (P) and the *aloe vera*-supplemented group (A), compared to the control group (C) (*p* < 0.05). Conversely, serum MDA levels (Figure 3c) were significantly reduced in the P and A groups relative to the control group (*p* < 0.05).

### 3.4 DNA and protein concentration

**Figure 4:**
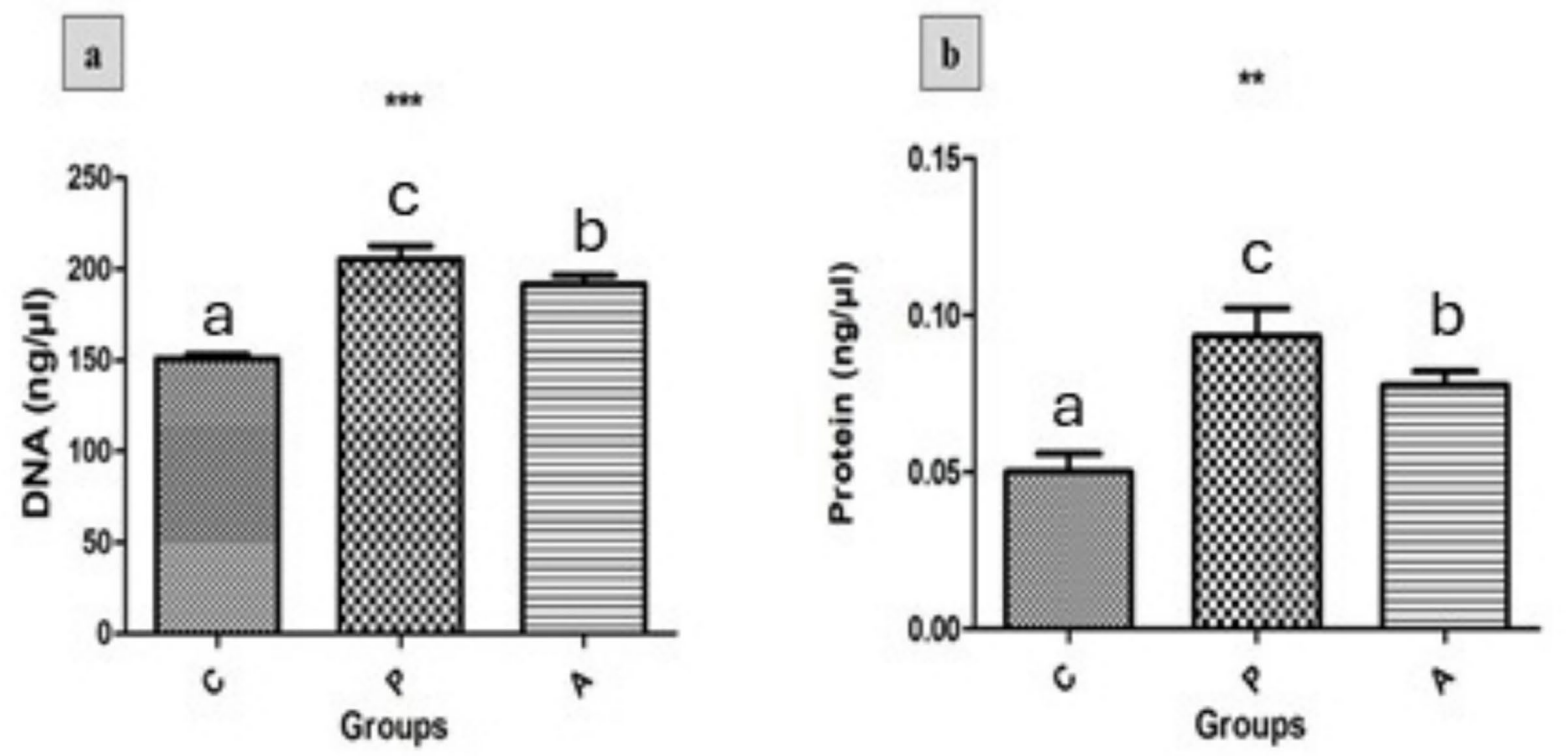
The effect of treatments on **(a)** DNA concentration and **(b)** protein concentration in growing rabbits across experimental groups. Group C = control; Group P = pomegranate; Group A = *Aloe vera*, *n* = (4). Asterisks denote significant differences compared to the control group (*p < 0.05, **p < 0.01, ***p < 0.001). Different letters indicate significant differences between experimental groups (*p* < 0.05).

A significant increase in DNA (Figure 4a) and protein concentrations (Figure 4b) was observed in the (P) and (A) groups compared to the control (*p* < 0.05). Furthermore, the (P) group exhibited higher levels of both DNA and protein than the (A) group.

### 3.5 Histological evaluation

**Figure 5:**
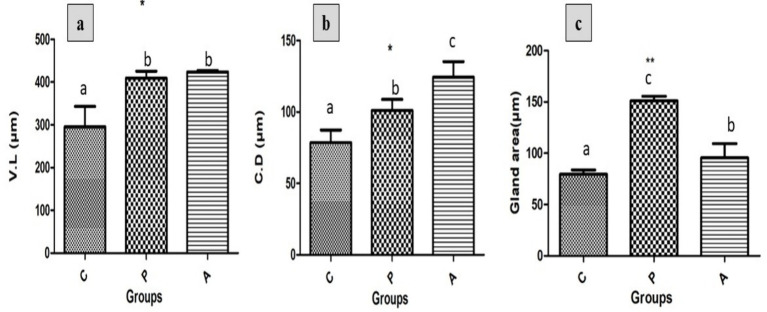
The effects of different treatments on **(a)** Villus length, **(b)** crypt depth, and **(c)** glandular area in growing rabbits across experimental groups. Group C = Control; Group P = Pomegranate; Group A = *Aloe vera*, *n* = (4). Asterisks denote significant differences compared to the control group (*p < 0.05, **p < 0.01, ***p < 0.001). Different letters indicate significant differences between experimental groups (*p* < 0.05).

Microscopic morphometric analysis of tissue sections from all experimental groups revealed a significant increase in villus length (Figure 5a), crypt depth (Figure 5b), and glandular area (Figure 5c) in both the (P)- and (A)-supplemented groups compared to the control. Notably, the (A) group exhibited slightly greater villus length and crypt depth than the (P) group, whereas the glandular area was marginally higher in the (P) group than in the (A) group.

### 3.6 Molecular docking simulation

**Figure 6:**
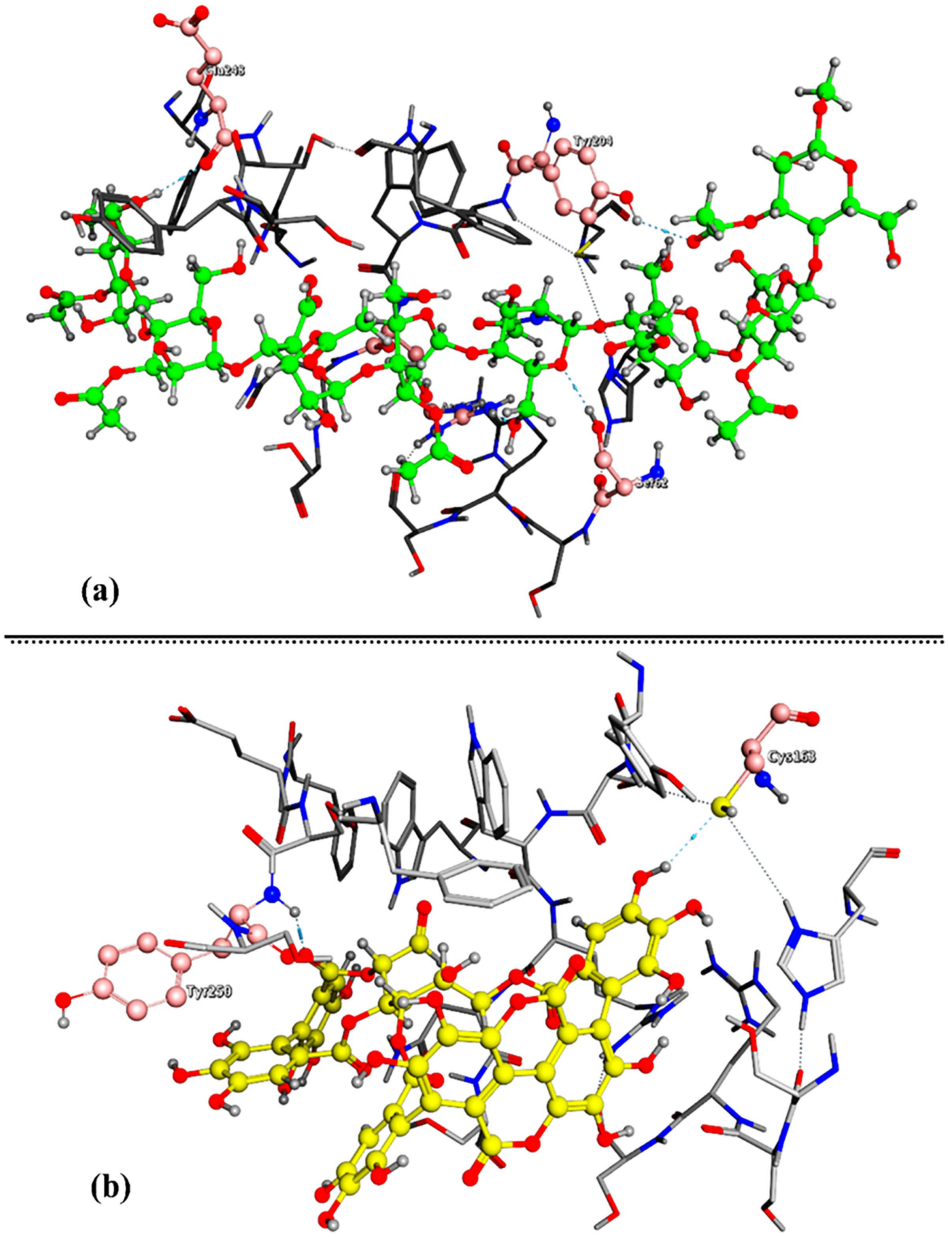
The output of the molecular docking simulation, the 3D representation of the best conformers’ complexation interactions between (**a**: Acemannan) and (**b**: Punicalagin) with BAX protein (ID: G1SQZ2).

Acemannan, a bioactive polysaccharide from *Aloe vera*, exhibits various biological activities, including immunomodulation, oral wound healing, periodontium regeneration, antitumor effects, and antigenotoxic properties, promoting immune cell activation (3). On the other hand, the principal bioactive compound in pomegranate peel, punicalagin, demonstrates potent antioxidant, cardioprotective, and lipid-lowering activities. Punicalagin increases the expression of key cholesterol metabolism regulators such as PPARγ, ABCA1, and CYP7A1, leading to improved cholesterol homeostasis and bile acid metabolism. These effects contribute to its potential in managing lipid disorders and improving cardiovascular health. In the case of the BAX protein, Acemannan (Figure 6a) showed better complexation than Punicalagin, scoring −10.627 Kcal/mol compared to Punicalagin’s −7.540 Kcal/mol (Figure 6b).

**Figure 7:**
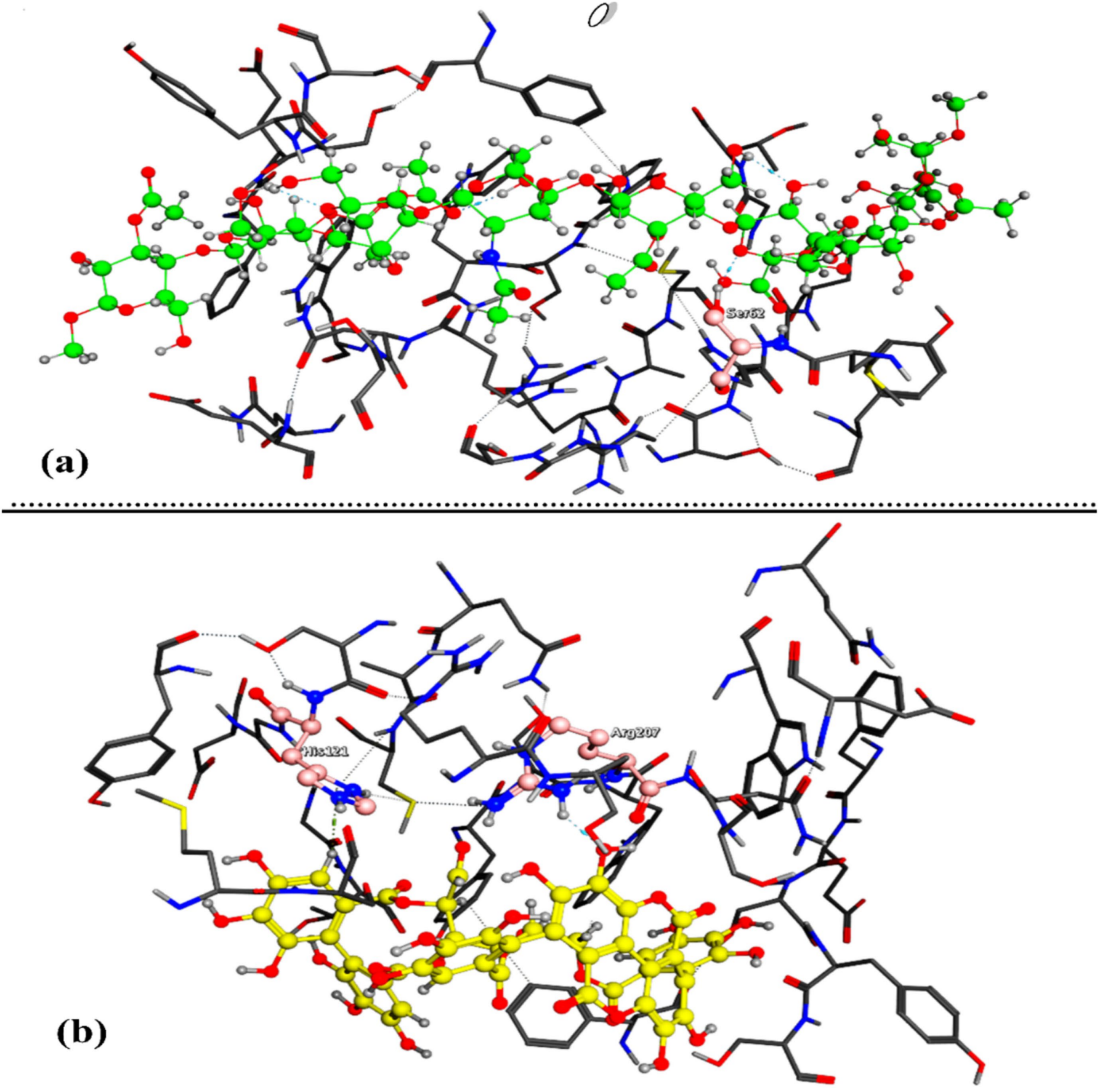
The output of the molecular docking simulation, the 3D representation of the best conformers’ complexation interactions between (**a**: Acemannan) and (**b**: Punicalagin) with SOD protein (coded: P41975).

In targeting the SOD protein (P41975), Acemannan demonstrated superior binding affinity compared to Punicalagin, yielding a docking score of −10.544 (Figure 7a) versus −7.663 (Figure 7b). This significant difference suggests that Acemannan facilitates a more stable complex reaction and possesses higher potential bioactivity.

Acemannan formed four intermolecular hydrogen bonds with BAX through the amino acids GLU248, TYR204, SER62, and ARG207 (Figure 6a) as shown in Table 2. In contrast, punicalagin formed two hydrogen bonds with BAX through the amino acids TYR250 and CYS163 (Figure 6b). In addition to the intramolecular hydrogen bond observed in both Acemannan and punicalagin, one intermolecular hydrogen bond resulted from the docked Acemannan with the SER62 amino acid (Figure 7a). Two intermolecular hydrogen bonds were formed from the docked punicalagin with SOD through the ARG207 and HIS121 amino acids (Figure 7b). Several types of non-covalent interactions also arise from complex interactions, including van der Waals forces and electrostatic attractions. This study offers an *in vitro* and *in silico* comparison of the bioactivity and complexation of Acemannan and punicalagin with BAX and SOD proteins.

**Table 2:** The output results of the best conformations obtained from the molecular docking simulation between both acemannan and punicalagin against BAX (ID: G1SQZ2) and SOD (ID: P41975).

Parameter	BAX (G1SQZ2)	
	Acemannan	Punicalagin
Score (Kcal/mol).	−10.627	−7.540
RMSD-refined	3.080	4.723
Hydrogen interactions	LIG H: GLU248 (O). LIG O: TYR204 (H). LIG O: SER 62 (H) LIG H: ARG 207 (O).	LIG O: TYR250 (H). LIG H: CYS163 (O).

Parameter	SOD (ID: P41975)	
	Acemannan	Punicalagin
Score (Kcal/mol).	−10.544	−7.663
RMSD-refined	3.795	2.467
Hydrogen interactions	LIG O: SER62 (H).	LIG O: ARG207 (H). (LIG H:HIS 121) (benzene ring).

## 4 Discussion

The weaning period is a critical and stressful stage in the mammalian life cycle. Maintaining intestinal integrity is essential for establishing a beneficial microbiota, promoting health, and ensuring overall well-being. Additionally, the valorization of natural by-products such as pomegranate peel and *Aloe vera* offers an economical and environmentally sustainable approach to enhancing growth and intestinal function in rabbits. In this study, we evaluated the effects of these phytogenic additives on intestinal health, metabolic activity, antioxidant capacity, and gastrointestinal morphology.

Our results demonstrate that supplementation with pomegranate peel and *Aloe vera* significantly improved body weight gain, serum metabolic parameters, digestive enzyme activity, antioxidant status, DNA and protein concentrations, and intestinal morphometry compared to the control group. These findings are consistent with previous studies showing that pomegranate peel supplementation enhances growth performance, feed efficiency, and beneficial gut microbial populations in rabbits (31), while *Aloe vera* supplementation has been associated with improved feed conversion, digestibility, and carcass quality in rabbits, broilers, and fish (32–34). The improvements observed in our study may be attributed to the high content of bioactive compounds, such as proanthocyanidins in pomegranate peel, which exert prebiotic effects, and mannan oligosaccharides in *Aloe vera*, which stimulate gut development and microbial balance.

Blood biochemical parameters indicated elevated serum protein, lipid, and glucose levels in the treated groups. The moderate increase in glucose, within physiological ranges, may be associated with adaptive metabolic responses during the post-weaning growth phase rather than a pathological alteration. This is consistent with previous reports that pomegranate peel extract enhances protein metabolism and antioxidant enzyme activities (GPx, SOD, CAT) (31, 35), while *Aloe vera* elevates serum protein and albumin levels and stimulates immune responses in poultry and rabbits (36, 37). The antioxidant effects of both supplements likely underpin these improvements. Pomegranate peel, rich in phenolic compounds, enhances antioxidant defense by scavenging free radicals and modulating apoptotic signaling pathways (e.g., upregulating Bcl-2 while downregulating Bax and caspase-3) (38, 39). Similarly, *Aloe vera* contains multiple antioxidant-active constituents (acemannan, aloin, aloe-emodin, and aloesin) that act synergistically to normalize redox balance, suppress lipid peroxidation, and modulate inflammatory mediators such as TNF-α and COX-2 (40, 41).

Morphological analyses revealed increased villus height, crypt depth, and glandular area in the duodenum of supplemented rabbits, indicating enhanced absorptive capacity and epithelial turnover. The Alcian blue staining highlighted increased mucin production in the pomegranate group, indicating improved mucosal protection. These observations align with earlier reports that phytogenic antioxidants preserve intestinal mucosa from oxidative stress and pathogens, thereby reducing gastrointestinal disturbances (42, 43). Elevated DNA concentrations in duodenal tissue further suggest enhanced cellular proliferation and intestinal development, likely to result from reduced oxidative DNA damage. Although we did not measure molecular markers directly, our findings are supported by recent studies demonstrating that pomegranate and *Aloe vera* modulate the expression of genes related to antioxidant defense (e.g., GPx, SOD), apoptosis (Bcl-2, Bax, caspase-3), and gut development (31, 38, 44). Furthermore, natural antioxidants can increase the reproduction of animals (45, 46).

Molecular docking analysis provided molecular level evidence supporting the *in vivo* antioxidant and cytoprotective outcomes of the present study. Both acemannan the principal bioactive polysaccharide in *Aloe vera* and punicalagin the predominant ellagitannin in pomegranate peel demonstrated strong binding affinities with two key regulatory proteins: the pro-apoptotic BAX and the antioxidant SOD. Acemannan exhibited higher binding energies toward both BAX (−10.627 kcal/mol) and SOD (−10.544 kcal/mol) compared to punicalagin (−7.540 vs. −7.663 kcal/mol, respectively), indicating a greater tendency to form stable ligand protein complexes and suggesting superior bioactivity.

Within the BAX docking complex, acemannan established multiple hydrogen bonds with essential amino acid residues such as GLU248, TYR204, and SER62, which are crucial for BAX activation and mitochondrial translocation. By occupying these residues, acemannan likely stabilizes the inactive conformation of BAX, thereby preventing mitochondrial outer membrane permeabilization and cytochrome c release critical events in the intrinsic apoptosis pathway (47, 48). Similar structural stabilization of BAX has been reported for polysaccharides with anti-apoptotic activity in hepatic and intestinal tissues (49). Conversely, punicalagin displayed moderate binding via TYR250 and CYS163 residues, suggesting partial inhibition of BAX oligomerization but a weaker interaction network overall.

In the SOD docking model, acemannan exhibited strong hydrogen bonding with ARG207 and SER62 within the enzyme’s active site, potentially enhancing catalytic stability and electron transfer efficiency. These interactions could aid in the conversion of superoxide radicals into hydrogen peroxide and oxygen, which aligns with the observed increase in TAC and CAT activities *in vivo* (50). Punicalagin also demonstrated favorable binding with SOD, interacting with polar and aromatic residues that support its SOD-mimetic and redox-regulatory functions (51). This molecular evidence supports the biochemical findings of reduced MDA concentrations and improved enzymatic antioxidant defense in the treated rabbits.

The superior binding efficiency of acemannan to both BAX and SOD reflects its dual role in mitigating oxidative stress and suppressing apoptosis, thereby protecting enterocytes and enhancing tissue regeneration (52). These effects may also be mediated through Nrf2 activation and subsequent upregulation of antioxidant genes, as reported in recent *in vitro* and *in vivo* studies on *Aloe vera* polysaccharides (53, 54). Similarly, punicalagin’s capacity to modulate Nrf2 and NF-κB signaling has been documented in animal models of oxidative injury, providing additional molecular support for its antioxidant and anti-inflammatory actions (55, 56).

Although molecular docking analysis revealed that acemannan exhibited stronger binding affinities toward both the pro-apoptotic BAX and the antioxidant SOD proteins compared with punicalagin, the in vivo findings demonstrated that most biochemical and physiological parameters were more markedly improved in the pomegranate peel treated group. This apparent discrepancy can be explained by several physiological and pharmacokinetic factors influencing the bioactivity of plant-derived compounds. Firstly, molecular docking provides a static prediction of ligand–protein interaction under idealized in silico conditions, whereas biological systems involve complex metabolic, enzymatic, and transport processes that modulate compound activity *in vivo* (52). Acemannan, being a high molecular-weight polysaccharide, has limited intestinal absorption and mainly exerts local effects on the gut mucosa (53). In contrast, punicalagin and its metabolites, including ellagic acid and urolithins, display higher bioavailability and can be absorbed systemically, reaching various organs and exerting broad antioxidant and anti-inflammatory actions (51). Secondly, the biological efficacy of pomegranate peel extract is not restricted to punicalagin alone. It contains multiple synergistic bioactive such as tannins, flavonoids, and anthocyanins, which collectively enhance digestive enzyme secretion, lipid metabolism, and antioxidant enzyme expression (55, 56). This synergism may explain the stronger improvements in body weight gain, serum protein, and digestive enzyme activity observed in the pomegranate-supplemented group.

Moreover, punicalagin interacts with gut microbiota, promoting the growth of beneficial bacteria and increasing the production of short-chain fatty acids (SCFAs), which improve nutrient absorption and intestinal health (57). Acemannan, though possessing stronger molecular binding, acts primarily through stabilization of intestinal epithelial cells and enhancement of local antioxidant defenses rather than systemic metabolic modulation (50). Finally, the two compounds may target different cellular pathways. Acemannan predominantly modulates apoptotic and oxidative stress pathways via BAX/SOD and Nrf2 signaling (47), while punicalagin exerts a wider influence, affecting NF-κB, MAPK, and AMPK pathways linked to digestion, immunity, and growth performance (52, 58). The present findings are consistent with previous reports about natural products (59, 60, 61). SOD, an essential antioxidant enzyme for detoxification in rabbits, was docked with acemannan and punicalagin. Acemannan showed a superior binding affinity (−10.544 kcal/mol) compared to punicalagin (−7.663 kcal/mol). These results align with Gouda et al. (62), who reported a binding energy of −3.33 kcal/mol for the betaine-SOD complex, suggesting that acemannan forms a more stable interaction with the enzyme.

## 5 Conclusion

The study presents comprehensive evidence that dietary supplementation with *Aloe vera* gel and pomegranate peel has significant positive effects on the growth performance, metabolic activity, and intestinal integrity of growing rabbits. Biochemical analyses showed that both supplements improved metabolic efficiency by enhancing plasma glucose, total protein and lipid metabolism, indicating improved nutrient utilization and energy balance. Increased digestive enzyme activities, such as amylase, lipase, and protease, further support the beneficial impact of these phytogenic additives on gastrointestinal function and nutrient absorption. Moreover, these compounds improved antioxidant status (TAC and SOD) and significantly lowered lipid peroxidation (MDA) in rabbit serum indicating antioxidant activity.

Molecular docking analysis demonstrated that acemannan and punicalagin exhibited strong binding affinities toward pro-apoptotic BAX (−10.627 and −7.540 kcal/mol, respectively) and antioxidant SOD (−10.544 and −7.663 kcal/mol, respectively) proteins. The stronger binding of acemannan suggests superior anti-apoptotic and antioxidant potential through stabilization of BAX and enhancement of SOD activity. Future studies should focus on clarifying the gene-level expression of apoptosis and antioxidant-related markers such as BAX, Bcl-2, SOD, CAT, and Nrf2. Additionally, exploring the dose-dependent and long-term effects of these supplements in different livestock species is crucial. These investigations will enhance the applicability of these findings and aid in the development of environmentally friendly and health-promoting feed strategies that support sustainable animal production and welfare.

## Data Availability

The raw data are available from the corresponding author upon reasonable request. Requests to access these datasets should be directed to ss.soliman@nrc.sci.eg.

## Footnotes

1
https://www.uniprot.org

2
https://pubchem.ncbi.nlm.nih.gov/
